# Analysis of microbiotas between traumatic and ulcerative wound: insights into challenges of current wounds managements

**DOI:** 10.3389/fcimb.2025.1622552

**Published:** 2025-09-02

**Authors:** Cheng Chen, Jiao Wang, Yifei Li, Zhenxin Fan, Yan Dai

**Affiliations:** ^1^ Wound Care Center, West China Hospital, West China School of Nursing, Sichuan University, Chengdu, Sichuan, China; ^2^ Key Laboratory of Bioresources and Eco-Environment of Ministry of Education (MOE), College of Life Sciences, Sichuan University, Chengdu, Sichuan, China; ^3^ Key Laboratory of Birth Defects and Related Diseases of Women and Children of MOE, Department of Pediatrics, West China Second University Hospital, Sichuan University, Chengdu, Sichuan, China

**Keywords:** wound, microbial composition, traumatic wound, ulcerative wound, outcome

## Abstract

**Objective:**

Wounds frequently occur during daily activities and clinical treatments, exerting substantial impacts on patients’ health status and recovery processes.

**Methods:**

All the patients were enrolled for this analysis from January 2022 to December 2022 who received wound management at Wound Care Center, West China Hospital, Sichuan University, which was a single center, prospectively designed observational study. In this investigation, microbial samples were collected from 106 traumatic wounds and 157 ulcerative wounds and analysed by 16S rRNA gene sequencing.

**Results:**

The findings revealed distinct differences in the microbial composition and function between traumatic and ulcerative wounds. Specifically, ulcerative wounds exhibited significantly higher abundances of infection- and pro-inflammation-related microbes, such as *Corynebacterium auriscanis*, *Finegoldia magna*, and *Corynebacterium striatum*, whereas traumatic wounds had a significantly elevated abundance of *Bifidobacterium longum*, which is known to promote wound healing.​ Regarding functional profiles, the relative abundances of lipopolysaccharide (LPS) biosynthesis, fatty acid biosynthesis, and metabolism of xenobiotics by cytochrome P450 were notably higher in the traumatic wound group. In contrast, the ulcerative wound group showed significantly greater relative abundances of drug metabolism - other enzymes, folate biosynthesis, and cysteine and methionine metabolism.

**Conclusion:**

Comparative analyses of the microbial communities in wounds reveled that traumatic wounds, especially non-postoperative wounds, were more easily to heal, however, the ulcerative wounds, especially the non-venous wounds, were more difficult to heal. These results suggest that different types of wounds harbor distinct microbial compositions, providing valuable data to inform improved clinical wound management strategies.

## Introduction

A wound is an injury that disrupts the normal anatomical structure and function of the skin with or without inner tissues, which can easily occur in daily life and is accompanied by some surgical medical treatments. Wounds can be classified into traumatic wounds and ulcerative wounds based on the type of damage to the surface. Traumatic wounds mainly include traumatism caused by sharp objects like knives or glass, burn wounds, surgical incisions, bites/scratches inflicted by pets or wild animals. Ulcerative wounds mainly include diabetic foot ulcers, venous ulcers, and arterial ulcers, among others. Wounds lead to the disruption of the skin barrier and can host a diverse range of microorganisms and pathogens (Legendre et al., 2002; Powers et al., 2016). Thus, their healing occurs in the backdrop of microbial communities, including bacteria, fungi, and viruses (White and Grice, 2023). Consequently, the advancement of wound treatment is of paramount importance.

In the wound, Microorganisms play a dual role in wound healing; there are both harmful and beneficial microorganisms. For example, the genus *Corynebacterium* has been proven to be a causative agent of significant infections, with its strains isolated from wounds and abscesses (Bernard, 2012). *C. striatum* shows a strong correlation with ulcerative wounds, such as foot ulcer, venous leg ulcer, and decubitus ulcer (Smith et al., 2010; Virgilio et al., 2023). Furthermore, the abundance of anaerobic bacteria was found to be positively correlated with wound ulcer depth (Gardner et al., 2013). For example, anaerobic bacteria *Anaerococcus* and *Finegoldia* (*F. magna*) have also been widely identified in pressure ulcers and may be associated with the stagnation or deterioration of wound healing (Dunyach-Remy et al., 2021; Travis et al., 2023). *F. magna* has been proven to induce inflammation through the activation of neutrophils (Neumann et al., 2020). Additionally, the leg wounds of patients suffering from venous ulcers are also colonized by bacteria, including anaerobic species, *Staphylococcus aureus*, *Pseudomonas aeruginosa*, *Corynebacterium*, *Finegoldia*, all of which are commonly identified in other types of ulcer wounds (Byeon et al., 2021; White and Grice, 2023). Besides, various types of skin wounds and related infections have been reported to be associated with *Staphylococcus aureus*, which causes most skin and soft tissue infections (Olaniyi et al., 2017) and was found to contribute to diabetic foot ulcer wounds and prevent functional recovery (Citron et al., 2007; White and Grice, 2023).

Inversely, some microorganisms can promote wound healing through various means, such as directly producing antibacterial substances to inhibit the proliferation of pathogens or preventing the adhesion of pathogens, and stimulating the body to produce various immune defensive substances (Dong et al., 2024). *Lactobacillus* and *Bifidobacterium* are widely studied probiotics that have also been found to play important probiotic roles in wound healing (Menni et al., 2023). For instance, *Bifidobacterium longum*, *Lactiplantibacillus plantarum*, and *Lactobacillus rhamnosus* have been shown to promote wound healing when applied topically, via strong anti-inflammatory activity and the promotion of healing and angiogenic factors expression (Panagiotou et al., 2023). Consequently, it is crucial to reveal the composition and function of the wound microbial community and uncover whether different types of wound microbiomes differ, as it may enhance our ability to manage and promote wound healing.

In the present study, a cohort consisting of 263 wound swabs was collected for revealing (1) the composition and function of the microbiome of different type of wounds (traumatic and ulcerative wounds); (2) the differences in microbial composition and function between traumatic and ulcerative wounds, and further in different type of traumatic wounds (postoperative and non-postoperative wounds) and ulcerative wounds (venous and non-venous ulcer wounds); (3) the potential impact of microorganisms on wound healing. Our results provided insightful data for a better understanding of the microorganisms related to different wound types and might help to better manage and treat wounds.

## Materials and methods

### Participants’ recruitment and sample collection

All the patients were enrolled for this analysis from January 2022 to December 2022 and received wound management at Wound Care Center, West China Hospital, Sichuan University, which was a single-center, prospectively designed observational research. The inclusion/exclusion criteria for sample collection were as follows: (1) age from 18 to 75 years old; (2) not in pregnancy or lactation; (3) no use of steroids or immunosuppressants within 2 weeks; (4) exclusion of cancerous or secondary hemorrhagic wounds. Consecutive patients with traumatic or ulcerative wounds had been enrolled, and a total of 263 wound samples were collected, including 106 from traumatic wounds and 157 from ulcerative wounds. Traumatic wounds include acute wounds such as incised wounds from sharp instruments, surgical incisions, animal bites, burns, and scalds (Lazarus et al., 1994). Ulcerative wounds consist of ulcerative lesions, such as chronic wounds, including diabetic foot, venous ulcers, arterial ulcers, and pressure ulcers (Lazarus et al., 1994; Powers et al., 2016). Before sample collection, the wound was rinsed twice with normal saline for cleansing, followed by disinfection of the periwound skin twice with povidone-iodine antiseptic solution to eliminate potential contaminants. Then, expressed the exudate from the wound bed and used three sterile swabs to soak up the exudate and store them in sterile tubes. Following collection, specimens were transferred and stored at -80°C within 15 minutes to maintain microbiological integrity for subsequent analytical procedures. The Ethics Committee of the West China Hospital of Sichuan University approved this study (2020No.239). The basic information of the project and the requirements for sample collection were provided to the patients by professional nurses. After the patients agreed to participate in the project, all the involved patients signed the written informed consent form before sampling.

### 16S rRNA gene sequencing

Total DNA from samples was extracted utilizing the QCTAB/SDS method. DNA concentration and purity were monitored on 1% agarose gels. After the quality inspection, DNA was diluted to 1 ng/μL using sterile water. Amplicon PCR targeted the V3–V4 region of the 16S rRNA gene using the 341F/806R primer (341F: 5'-CCTAYGGGRBGCASCAG-3', 806R: 5'-GGACTACNNGGGTATCTAAT-3') with the barcode. All PCR reactions contained 15 µL of Phusion^®^ High-Fidelity PCR Master Mix (New England Biolabs), 0.2 µM of each primer and 10ng target DNA, and cycling conditions consisted of a first denaturation step at 98°C for 1 min, followed by 30 cycles at 98°C (10s), 50°C (30s) and 72°C (30s) and a final 5 min extension at 72°C. Mix an equal volume of volume of 1X loading buffer (contained SYB green) with PCR products and perform electrophoresis on 2% agarose gel for detection. PCR products were mixed in equidensity proportions. Then, the mixture PCR products was purified with Qiagen Gel Extraction Kit (Qiagen, Germany). Sequencing libraries were generated by NEBNext^®^ Ultra™ IIDNA Library Prep Kit (Cat No. E7645) following the manufacturer’s recommendations, and index codes were added. The library quality was assessed on the Qubit@ 2.0 Fluorometer (Thermo Scientific) and Agilent Bioanalyzer 2100 system. At last, the library was sequenced on an Illumina NovaSeq platform and 250 bp paired-end reads were generated.

### Data processing

Paired-end reads were assigned to samples based on their unique barcode and truncated by cutting off the barcode and primer sequence. Paired-end reads were merged using FLASH (VI.2.7, http://ccb.jhu.edu/software/FLASH/) (Magoc and Salzberg, 2011), which was designed to merge paired-end reads when at least some of the reads overlap the read generated from the opposite end of the same DNA fragment. The splicing sequences were called raw tags. Quality filtering on the raw tags was performed under specific filtering conditions to obtain the high-quality clean tags (Bokulich et al., 2013) according to the FASTP. The UCHIME algorithm (UCHIME Algorithm, http://www.drive5.com/usearch/manual/uchime_algo.html) was employed to compare the raw tags with the reference Silva database (v138.1, https://www.arbsilva.de/) (Robeson et al., 2021), aiming to detect and remove chimera sequences. The QIIME2 (v2020.11.1) (Hall and Beiko, 2018) pipeline with default parameters was used for data analysis. Specifically, DADA2 was applied for filtering noise sequences, correcting edge sequence errors, removing chimeric sequences and accidental sequences, thereby obtaining a high-resolution analogue of the operational taxonomic unit (OTU) table. Subsequently, the scikit-learn method was adopted to assign and classify taxonomy against the Silva database with a 99% similarity threshold.

### Statistical analysis for data of 16S rRNA gene data

The variations in alpha diversity between groups were compared using the t-test. Linear discriminant analysis (LDA) Effect Size (LEfSe) was used to reveal intergroup differences in microbial communities, with the screening criteria LDA > 3, *P* < 0.05 (Chang et al., 2022). The microbial functional differences between groups were performed and visualized by STAMP v2.1.3 (Parks et al., 2014). The Principal Co-ordinates Analysis (PCoA) was tested by PERMANOVA (permutations=999) using the adonis2 function in vegan (v2.6.4) (Dixon, 2003). Graphical visualization used R packages including ggplot2 v3.4.1 (Wickham, 2016), ggpubr v0.6.0 (Kassambara, 2023), ggprism v1.0.4 (Dawson, 2024), permute v0.9.7 (Simpson, 2022), dplyr v1.1.0, phyloseq v1.40.0 (McMurdie and Holmes, 2013).

## Results

### Sample information

To elucidate the composition and structure of the microbial community in wounds and its influence on wound healing, we collected microbial samples from various types of wounds and conducted 16S rRNA gene sequencing. A total of 263 wound samples were obtained, including 106 traumatic and 157 ulcerative wound samples. Traumatic wounds include home pet bite injuries, surgical incisions, burns, and scalds, while ulcerative wounds primarily encompass venous ulcers, scar ulcers, immune-related ulcers, diabetic foot ulcers, and radiation-induced ulcers.

### Microbiotas composition of traumatic and ulcerative wound

**Figure 1:**
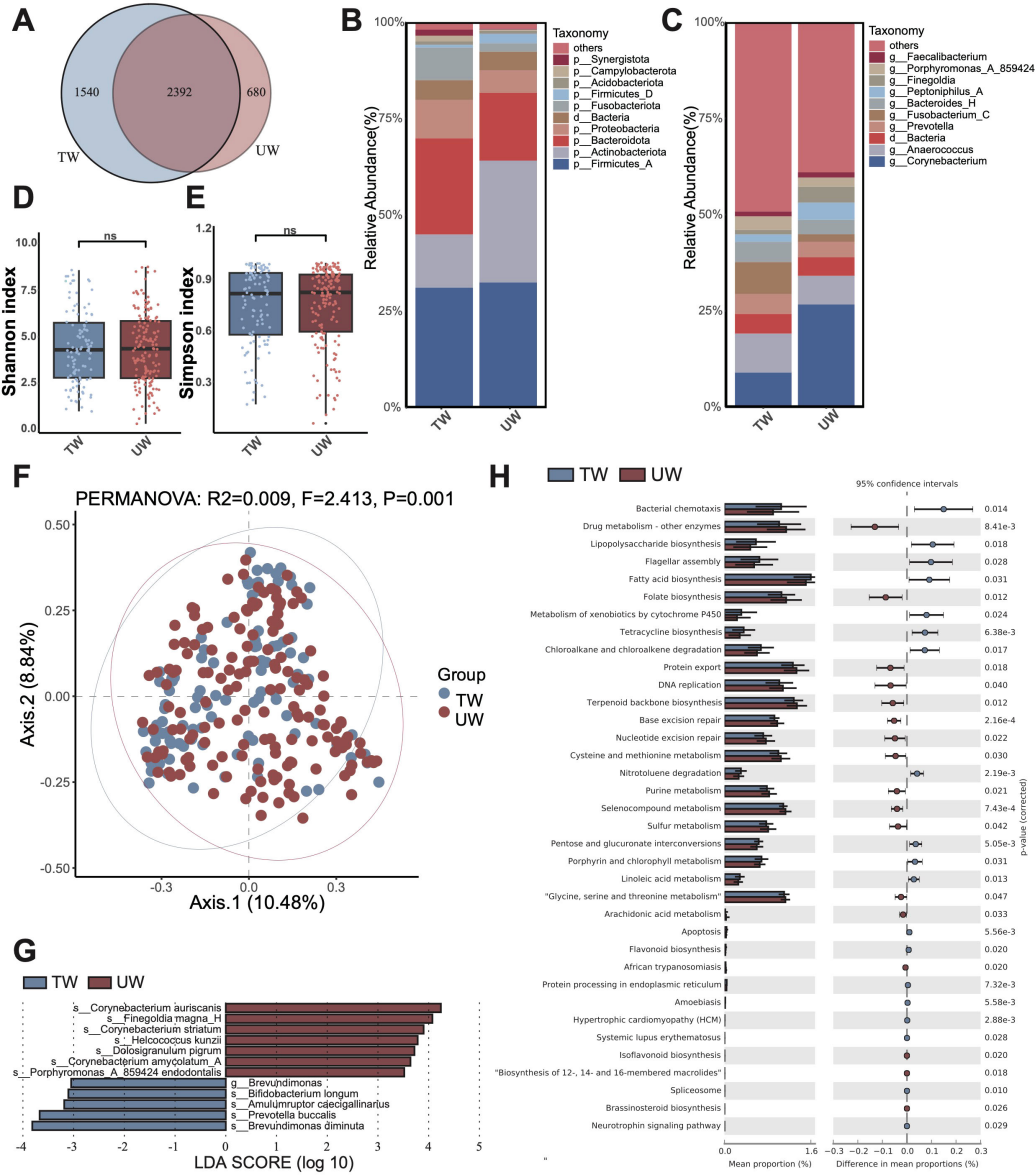
Microbiota composition and differences between traumatic wounds (TW) and ulcerative wounds (UW). **(A)** The number of unique OTUs and shared OTUs in the TW group and the UW group. **(B)** The microbial composition at the phylum level. The “others” refers to the overall relative abundance of all other phyla that are not included in the top 10 displayed microorganism groups. **(C)** The microbial composition at the genus level. The **(D)** Shannon diversity index and **(E)** Simpson diversity index of the TW group and UW group. A t-test was used to compare the two groups. “ns” indicates no significant difference, meaning *P* value > 0.05. **(F)** The PCoA between the TW group and the UW group. **(G)** The differential microbiota species between the TW and the UW wounds, using LEfSe with *P* < 0.05 (Kruskal-Wallis rank sum test) and the absolute values of LDA >3. **(H)** The composition and difference of microbial function between the TW and the UW wounds.

We first revealed the composition of the microbial community and its differences between traumatic and ulcerative wounds. Initially, we characterized the microbial OTUs present in the wound samples. The traumatic group exhibited a total of 3,072 OTUs, while the ulcerative group revealed 3,932 OTUs. Notably, 2,392 OTUs were shared between the two groups, however, 680 OTUs were exclusive to the traumatic group and 1,540 OTUs were exclusive to the ulcerative group (**Figure 1A**). The exclusive microbiota in the traumatic group included *Truepera radiovictrix*, *Cnuella takakiae*. In contrast, the exclusive microbiotas of the ulcerative group comprised *Prevotella denticola*, *Bulleidia extructa*, which have been previously identified at the abscess site, and *Clostridium cadaveris*.

At the phylum level, the most abundant phyla in the traumatic group were Firmicutes_A, Bacteroidota, and Actinobacteriota. In contrast, the ulcerative group displayed a distinct composition with Firmicutes_A, Actinobacteriota, and Bacteroidota as its top three predominant phyla. Notably, the relative abundance of Actinobacteriota was increased in the ulcerative group compared to that in the traumatic group (**Figure 1B**). At the genus level, *Anaerococcus*, *Corynebacterium*, and *Fusobacterium*_C ranked as the top three genera within the traumatic group. Conversely, *Corynebacterium*, *Anaerococcus*, and an unannotated genus dominated in the ulcerative group. Importantly, an increase in *Corynebacterium* relative abundance was observed in the ulcerative group relative to that of the traumatic group (**Figure 1C**).

### Differences in microbiota composition and function between traumatic and ulcerative wounds

Firstly, diversity analysis revealed that the alpha diversity (Shannon and Simpson indices) of bacterial communities on traumatic and ulcerative wounds was not significantly different (**Figures 1D, E**). Furthermore, the beta diversity (PCoA) of bacterial communities showed significant yet slight differences between traumatic wounds and ulcerative wounds (**Figure 1F**).

Then, we performed Linear discriminant analysis Effect Size (LEfSe) to identify the differential microbiota between traumatic and ulcerative wounds (**Figure 1G**). As a result, we identified seven microbial species with significantly higher relative abundance in ulcerative wounds, and five species with significantly higher in traumatic wounds. In the ulcerative wound, the differential species were found associated with infection and pro-inflammatory reactions, such as *Corynebacterium auriscanis*, *Finegoldia magna*, and *Corynebacterium striatum*. Conversely, the differential species in traumatic wounds included *Brevundimonas diminuta*, a multidrug-resistant opportunistic pathogen, and *Bifidobacterium longum*, which has been found that can enhance wound healing by regulating inflammatory response, promoting angiogenesis and inducing tissue repair. The significantly higher relative abundance of *Bifidobacterium longum* in traumatic wounds implies that traumatic wounds might experience improved recovery.

Furthermore, we identified functional differences in microbiota between traumatic and ulcerative wounds (**Figure 1H**; **Supplementary Table S1**). We found that the relative abundance of lipopolysaccharide (LPS) biosynthesis, fatty acid biosynthesis, metabolism of xenobiotics by cytochrome P450, flagellar assembly, bacterial chemotaxis, and tetracycline biosynthesis were significantly higher in the traumatic wound group. In contrast, the relative abundance of drug metabolism-other enzymes, folate biosynthesis, cysteine and methionine metabolism, DNA replication, glycine, serine and threonine metabolism were significantly higher in the ulcerative wound group.

### Microbiota characteristics of different types of traumatic wounds

**Figure 2:**
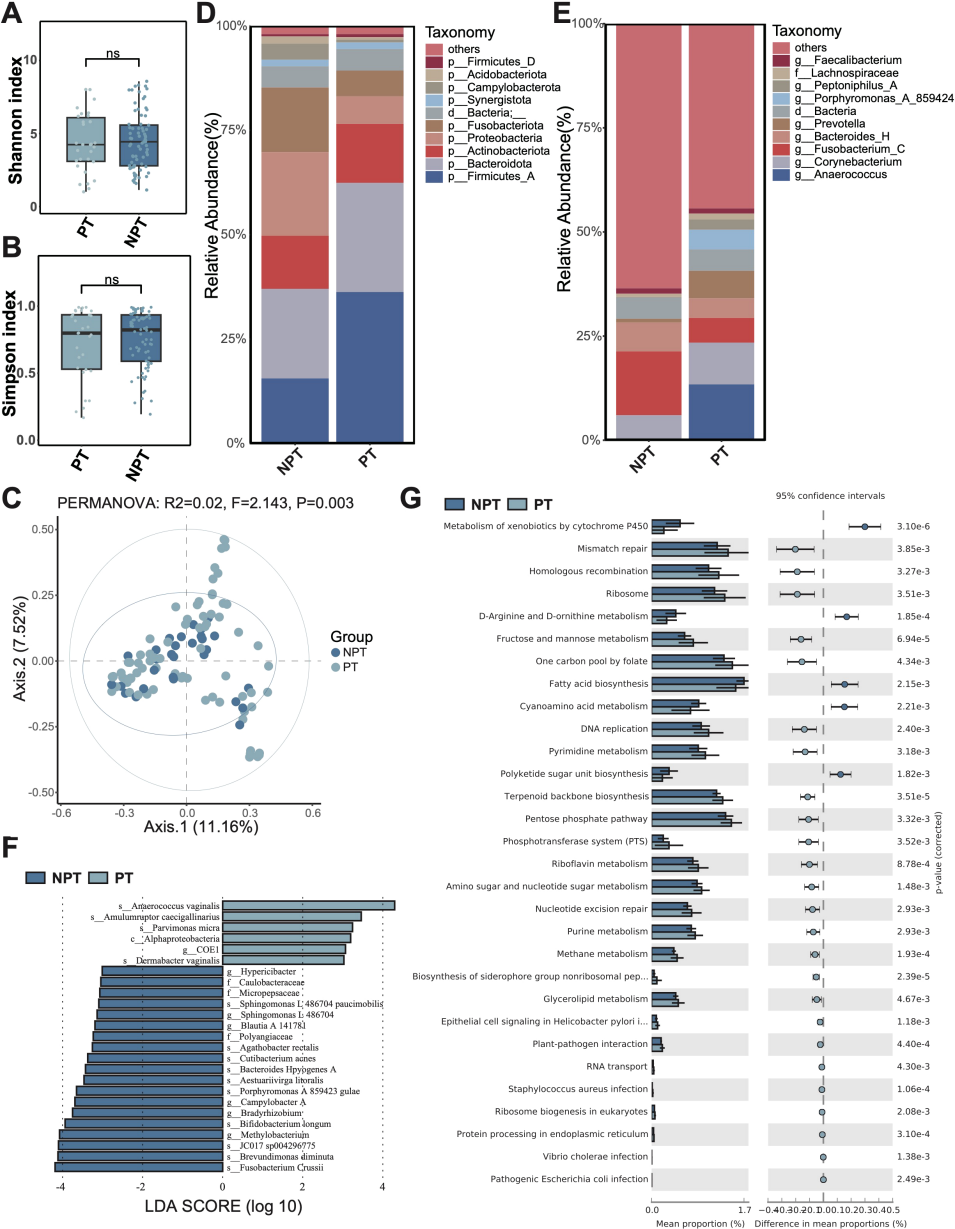
Microbiota composition and differences between postoperative traumatic wounds (PT) and non-postoperative wounds (NPT). **(A)** The Shannon diversity index and **(B)** the Simpson diversity index of the PT group and NPT group. A t-test was used to compare the two groups. “ns” indicates no significant difference, meaning *P* value > 0.05. **(C)** PCoA between the PT group and NPT group. **(D)** The microbial composition at the phylum level. **(E)** The microbial composition at the genus level. **(F)** The differential microbiota species between the PT group and NPT group, using LEfSe with *P* < 0.05 (Kruskal-Wallis rank sum test) and the absolute values of LDA >3. **(G)** The composition and difference of microbial function between the PT group and NPT group. The figure shows only the parts with *p-values* (corrected) < 0.01; for the complete results, see **Supplementary Table S2**.

Considering the different types of traumatic wounds, we compared the differences in microbiome composition and function between postoperative and non-postoperative wounds. Postoperative wounds refer to the surgical incision, while Non-postoperative wounds refer to any other traumatic wound except the surgical incision, e.g., trauma, burns, scalds, animal bites. Firstly, diversity analysis showed that there was no significant difference in α-diversity (Shannon and Simpson index) between postoperative wounds and non-postoperative wounds (**Figures 2A, B**). In addition, there was no significant difference in microbial β diversity (PCoA) between postoperative and non-postoperative wounds (**Figure 2C**).

Subsequently, we assessed the microbiota composition of postoperative and non-postoperative wounds. At the phylum level, Firmicutes_A, Bacteroidota, and Actinobacteriota were identified as the most abundant phyla in the postoperative wound group (**Figure 2D**). In contrast, the three most abundant phyla in the non-postoperative wound group were Bacteroidetes, Proteobacteria, and Firmicutes_A. Compared to the non-postoperative wound group, the relative abundance of Proteobacteria decreased in the postoperative wound group; conversely, the relative abundance of Firmicutes_A increased. At the genus level, *Anaerococcus*, *Corynebacterium*, and *Fusobacterium*_C were found to be the most abundant genera in the postoperative wound group, while *Fusobacterium*, *Bacteroides*_H, and *Corynebacterium* dominated in the non-postoperative group (**Figure 2E**). Notably, compared to the non-postoperative wound group, the relative abundance of *Fusobacterium*_C was reduced in the postoperative wound group, while the abundance of *Anaerococcus* increased significantly in the postoperative wound group.

The LEfSe analysis identified six and 19 microbiota species with significantly higher relative abundance in the postoperative and non-postoperative wound groups, respectively (**Figure 2F**). In the non-postoperative wound group, we noticed that many species with significantly higher relative abundance were pathogenic bacteria, such as *Brevundimonas diminuta*, *Cutibacterium acnes*, *Bacteroides Hpyogenes*, indicating a potential poor wound healing ability. However, *Bifidobacterium longum* also showed significantly higher relative abundance in the non-postoperative wound group. In the postoperative wound group, we also identified one abscesses-related species with significantly higher relative abundance, *Parvimonas micra*.

Furthermore, the functional difference analysis showed that various amino acid metabolism (D-arginine, D-ornithine, phenylalanine), and metabolism of xenobiotics by cytochrome P450, fatty acid biosynthesis, polyketide sugar unit biosynthesis, cyanoamino acid metabolism, etc. displayed significantly higher relative abundance in the non-postoperative wound group (**Figure 2G**; **Supplementary Table S2**). Some bacterial infection pathways (*Staphylococcus aureus*, *Vibrio cholera*, and *Escherichia coli*), and saccharometabolism pathways (e.g. glycolysis/gluconeogenesis, starch and sucrose metabolism, and fructose and mannose metabolism), and terpenoid backbone biosynthesis, riboflavin metabolism, and other pathways showed significant higher relative abundance in the postoperative wound group.

### Microbiota characteristics of different types of ulcerative wound

**Figure 3:**
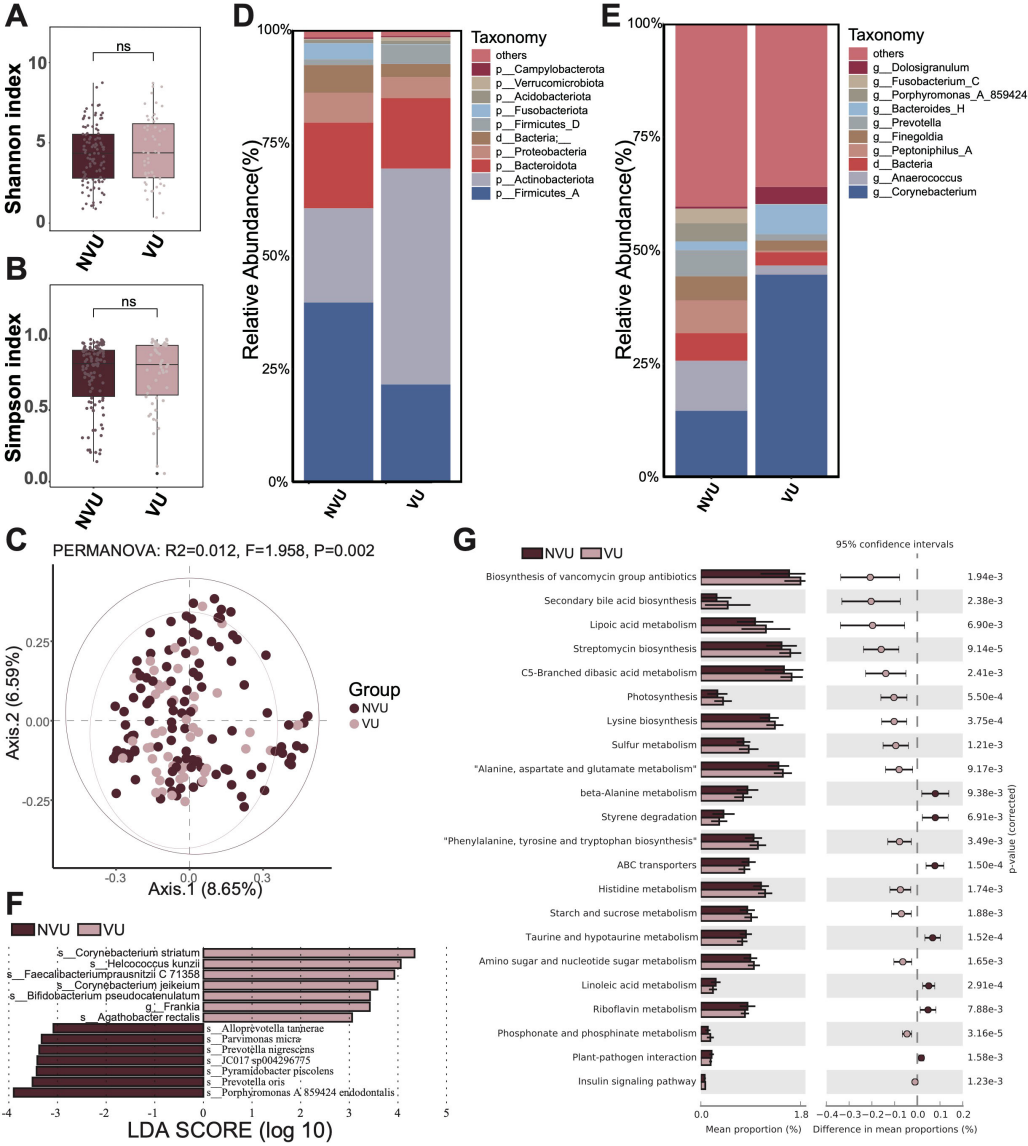
Microbiota composition and differences between venous ulcerative wounds (VU) and non-venous ulcerative wounds (NVU). **(A)** The Shannon diversity index and **(B)** the Simpson diversity index of the VU group and NVU group. A t-test was used to compare the two groups. “ns” indicates no significant difference, meaning *P* value > 0.05. **(C)** The PCoA between the VU group and NVU group. **(D)** The microbial composition at the phylum level. **(E)** The microbial composition at the genus level. **(F)** The differential microbiota species between the VU group and NVU group, using LEfSe with *P* < 0.05 (Kruskal-Wallis rank sum test) and the absolute values of LDA >3. **(G)** The composition and difference of microbial function between the VU group and NVU group. The figure shows only the parts with *p-values* (corrected) < 0.01; for the complete results, see **Supplementary Table S3**.

Meanwhile, we performed a comparative analysis between different types of ulcerative wounds, specifically venous and non-venous. The non-venous ulcerative wound contained cicatricial ulcer, radiation ulcer, immune ulcer, diabetic foot, gouty ulcer and others. Similarly, we observed no significant microbial diversity differences between venous and non-venous ulcerative wounds regarding both α diversity (Shannon and Simpson index, **Figures 3A, B**) and β diversity (PCoA, **Figure 3C**).

Then, we displayed the microbial composition of the venous and non-venous groups. At the phylum level, the three most abundant microbiota in the venous group were Actinobacteriota, Firmicutes_A, and Bacteroidota (**Figure 3D**). In contrast, the top three abundant microbiotas in the non-venous group were Firmicutes_A, Actinobacteriota, and Bacteroidota. The relative abundance of Firmicutes_A decreased in the venous group compared to that in the non-venous group, whereas Actinobacteriota increased. At the genus level, *Corynebacterium*, *Bacteroides*_H, and *Dolosigranulum* were identified as the most abundant genera in the venous group (**Figure 3E**). The top three abundant genera in the non-venous group were *Corynebacterium*, *Anaerococcus*, and *Peptoniphilus*_A. Notably, we noticed that the relative abundance of *Corynebacterium* increased in the venous group compared to that in the non-venous group.

Additionally, we conducted LEfSe to elucidate whether the two groups exhibit differential microbiota. In results, we identified seven differential microbial species in each group (venous and non-venous groups) with significantly higher relative abundance (**Figure 3F**). In the venous group, the microbiotas with significantly higher abundance included *Corynebacterium striatum*, *Helcococcus kunzii*, *Faecalibacterium prausnitzii* C 71358, *Corynebacterium jeikeium*, *Bifidobacterium pseudocatenulatum*, *Frankia* sp., *Agathobacter rectalis*. The non-venous group identified *Porphyromonas endodontalis* A 859424, *Prevotella oris*, *Pyramidobacter piscolens*, JC017 sp004296775, *Prevotella nigrescens*, *Parvimonas micra*, and *Alloprevotella tannerae* as differential species. We observed that several microbes in the venous group were associated with anti-inflammatory regulation, including *Faecalibacterium prausnitzii* and *Bifidobacterium pseudocatenulatum*, suggesting that venous ulcer wounds heal more readily than non-venous ulcer wounds.

Finally, we compared the differences in microbial function between venous and non-venous groups (**Figure 3G**; **Supplementary Table S3**). In the venous group, we found that pathways like streptomycin biosynthesis, lysine biosynthesis, photosynthesism, sulfur metabolism, amino sugar and nucleotide sugar metabolism, secondary bile acid biosynthesis, were significantly more abundant. In the non-venous group, pathways like ABC transporters, taurine and hypotaurine metabolism, linoleic acid metabolism, styrene degradation, riboflavin metabolism, beta-Alanine metabolism, were significantly more abundant.

## Discussion

Wounds occur frequently in life, including traumatic and ulcerative wounds. This study reveals that different types of wounds have microbial differences. Targeted treatment with specific microorganisms may help wounds heal better. Firstly, we revealed that the dominant compositions of bacteria in both traumatic and ulcerative wounds were pathogenic, suggesting a potential influence of microorganisms on wound healing. Suppurative and decay-related bacteria, such as *Prevotella denticola*, *Bulleidia extructa*, *Clostridium cadaveris*, were only found in ulcerative wounds. Moreover, *Corynebacterium auriscanis*, *Finegoldia magna*, and *Corynebacterium striatum*, which had been reported to be detected in suppurating and decay sites related to infection and inflammation (Delsing et al., 2014; Nakamura et al., 2018; Javeed et al., 2022; Abarca et al., 2023), showed significantly higher relative abundance in ulcerative wounds. The bacterium *C. striatum* was widely identified in the sampled wounds, closely related to foot ulcers, venous ulcers of lower limbs, etc., which could infect peripheral tissues and complicate wound healing (Virgilio et al., 2023), and might be an important pathogen in ulcerative wounds. In contrast, *Bifidobacterium longum* was identified as more abundant in traumatic wounds. This bacterium had been shown to promote wound healing via inflammation suppression (e.g., decrease in localized tissue edema, mast cell degranulation, and TNF-alpha release) (Yao et al., 2021; Menni et al., 2023; Panagiotou et al., 2023). Furthermore, both traumatic and ulcerative wounds were found to harbor certain pathogenic bacteria. However, ulcerative wounds were associated with a diverse range of abscess-related bacteria, whereas traumatic wounds presented a more favorable presence of probiotics. These findings demonstrated that the deleterious effects mediated by microbial colonization in traumatic wounds were less severe relative to those observed in ulcerative lesions. It also suggested that microbes might play a role in making ulcerative wounds more difficult to manage.

Moreover, we also found that postoperative wounds had fewer pathogenic bacteria than non-postoperative wounds in traumatic wounds. This could be attributed to the fact that postoperative wounds were made under sterile conditions, whereas non-postoperative wounds were prone to exposure to various environmental factors in daily life. Venous ulcers were the most common type of chronic lower limb ulcer and affect the health of many people (Millan et al., 2019). Comprehensive analyses have demonstrated that microbial community composition in chronic wounds—including diabetic foot ulcers, venous leg ulcers, pressure injuries (formerly decubitus ulcers), and non-healing surgical wounds—does not exhibit statistically significant wound type-specific patterns, suggesting common pathophysiological mechanisms may transcend etiological classifications (Wolcott et al., 2016). Similarly, we also found that the types of wounds rarely explained the difference in microbiome (**Figure 1F**, **2C**, **3C**). However, differences had been identified among the microorganisms in different types of ulcer wounds. Analysis revealed significant enrichment of anti-inflammatory bacterial species, notably *Faecalibacterium prausnitzii* and *Bifidobacterium pseudocatenulatum*, in venous ulcer microbiomes compared to non-venous ulcer wounds. This differential microbial signature, characterized by organisms known to produce secondary bile acids and modulate host immune response (Zha et al., 2024), suggests potentially enhanced healing capacity in venous ulcerations relative to other chronic wound etiologies, though causality remains to be established through interventional studies.

In addition, it had been frequently reported in studies that *Staphylococcus*, *Pseudomonas*, *Finegoldia*, *Corynebacterium*, and *Anaerococcus* were common bacteria in ulcerative wounds (Wolcott et al., 2016), but *Staphylococcus* and *Pseudomonas* had quite low abundance in our wounds. The microbiota with the highest relative abundance in our study were *Anaerococcus* and *Corynebacterium* in both traumatic and ulcerative wounds, indicating the diversity and differences of microorganisms in the wound. Therefore, different clinical treatment approaches, tailored to different types of wound pathogens, are highly necessary. However, this study also has certain limitations. This study only revealed the differences between traumatic wounds and ulcerative wounds in terms of microbial composition and functional annotations. Technically, 16S rRNA sequencing is inferior to metagenomic sequencing in terms of functionality and more in-depth analysis. Besides, there is a lack of research on the dynamic changes of microbiotas in the process of wound healing, and more in-depth research will be conducted in the future. Our results also require more in-depth microbial cultivation or animal experiments for verification.

In conclusion, we revealed the composition and function of the microbiome of traumatic and ulcerative wounds, and also compared their differences. In addition, the difference between postoperative and non-postoperative wounds, venous ulcer and non-venous ulcer was deeply compared and fully revealed the microbial difference of different types of wounds. Suppurative and decay-related bacteria such as *Prevotella denticola*, *Bulleidia extructa*, *Clostridium cadaveris* were only found in ulcerative wounds, and infection and inflammation-related bacteria *Corynebacterium striatum*, *Corynebacterium auriscanis*, and *Finegoldia magna*, have increased significantly in ulcer wounds. In traumatic wounds, *Bifidobacterium longum*, which promotes healing, was significantly increased. Our findings demonstrated that, compared to ulcerative wounds, the microbial community in traumatic wounds was more conducive to wound healing, and suggested that different types of wounds should be treated in different ways for certain microbiotas.

## Data Availability

The raw sequencing reads from this study have been submitted to the China National GeneBank Database (CNGBdb) with the project accession CNP0007326.
